# Inspiratory Muscle Training in Obstructive Sleep Apnea Associating Diabetic Peripheral Neuropathy: A Randomized Control Study

**DOI:** 10.1155/2020/5036585

**Published:** 2020-06-12

**Authors:** Samah A. Moawd, Alshimaa R. Azab, Saud M. Alrawaili, Walid Kamal Abdelbasset

**Affiliations:** ^1^Department of Physical Therapy for Cardiovascular/Respiratory Disorders and Geriatrics, Faculty of Physical Therapy, Cairo University, Giza, Egypt; ^2^Department of Health and Rehabilitation Sciences, College of Applied Medical Sciences, Prince Sattam bin Abdulaziz University, Alkharj, Saudi Arabia; ^3^Department of Physical Therapy for Pediatrics, Faculty of Physical Therapy, Cairo University, Giza, Egypt; ^4^Department of Physical Therapy, Kasr Al-Aini Hospital, Cairo University, Giza, Egypt

## Abstract

**Objective:**

This work is aimed at assessing the effects of inspiratory muscle training on lung functions, inspiratory muscle strength, and aerobic capacity in diabetic peripheral neuropathy (DPN) patients with obstructive sleep apnea (OSA).

**Methods:**

A randomized control study was performed on 55 patients diagnosed with DPN and OSA. They were assigned to the training group (IMT, *n* = 28) and placebo training group (P-IMT, *n* = 27). Inspiratory muscle strength, lung functions, and aerobic capacity were evaluated before and after 12 weeks postintervention. An electronic inspiratory muscle trainer was conducted, 30 min a session, three times a week for 12 consecutive weeks.

**Results:**

From seventy-four patients, 55 have completed the study program. A significant improvement was observed in inspiratory muscle strength (*p* < 0.05) in the IMT group while no changes were observed in the P-IMT group (*p* > 0.05). No changes were observed in the lung function in the two groups (*p* > 0.05). Also, VO_2_max and VCO_2_max changed significantly after training in the IMT group (*p* < 0.05) while no changes were observed in the P-IMT group (*p* > 0.05). Other cardiopulmonary exercise tests did not show any significant change in both groups (*p* > 0.05).

**Conclusions:**

Based on the outcomes of the study, it was found that inspiratory muscle training improves inspiratory muscle strength and aerobic capacity without a notable effect on lung functions for diabetic patients suffering from DPN and OSA.

## 1. Introduction

Diabetes mellitus (DM) is a global disease in which blood glucose level regulation is affected causing major health implications. The global epidemic recording of diabetes was about 400 million people in 2013 and expected to reach 600 million by 2035 [1, 2]. Type 2 diabetes (T2D) ranged more than 90% of all diabetic patients and it affects multiple body systems leading to cardiovascular, cerebrovascular, and renal complications particularly in poorly controlled diabetes and may cause death [3, 4]. Diabetic patients have a double risk for mortality than nondiabetics [5, 6].

More than 60% of diabetic patients are commonly suffering from nervous system damage, known as diabetic peripheral neuropathy (DPN) and defined as impairments in metabolic and microvessel functions due to chronic hyperglycemia and cardiovascular dysfunctions. These impairments are symmetrical and longstanding [7]. DPN affects approximately 30% of diabetic patients aged more than 40 [8].

Obstructive sleep apnea (OSA) is known as partial or complete episodes of upper airway obstruction during sleep. Commonly, episodic airway obstruction is associated with oxyhemoglobin desaturations and sleep arousals. OSA symptoms include chronic snoring, insomnia, gasping and breath-holding, unrefreshing sleep, and daytime sleepiness [9].

OSA is a frequent disorder characterized by apnea and hypopnea even with continued respiratory efforts and occurs for ten or more seconds because of a collapsed upper airway [10, 11]. This is habitually associated with sleep disturbances, arousals, and oxygen saturation impairments [12]. Episodes of upper airway obstruction during sleep, repetitive short interruptions of sleep, and impairment of oxyhemoglobin saturation are related to OSA [13, 14]. Symptoms of fatigue, excessive daytime sleepiness, and morning headaches occur due to activating the sympathoadrenal system, oxidative stress, systemic inflammation, and impaired adipokines. Subsequently, these symptoms result in an increased cardiovascular disease sustainability, higher blood pressure, and metabolic syndrome in diabetic patients [15, 16].

Lately, it has been reported that OSA and DPN are strongly correlated and OSA is prevalent in 23-87% of T2D [17] indicating that OSA and T2D are a risk factor for each other [6]. Besides, OSA negatively affects the treatment of T2D and may worsen preexisting OSA. The causes of the relationship between the OSA and PDN remain unknown [18]. It is a bidirectional association between OSA and T2D as OSA may aggravate and amplify T2D and subsequent complications such as DPN. Eventually, OSA may have contributed to T2D, resulting in a vicious circle. In addition, mechanisms including oxidative stress, inflammation, AGEs, and PKC signaling contributed to DPN and OSA. These underlying mechanisms may occur simultaneously in patients with T2D leading to DPN and OSA [19–21].

Only few studies have assessed the influences of inspiratory muscle training (IMT) in DPN with OSA [22], while many documents have reported the efficacy of this type of exercise on several medical issues [23, 24]. The effects of IMT on aerobic capacity and inspiratory muscle strength are debatable. Conducting the IMT resulted in improvements in inspiratory muscle strength and aerobic capacity in asthmatics [25], while another study found that IMT did not show any changes in aerobic capacity and lung functions in T2D [26]. Therefore, we aimed to assess the effects of IMT on lung functions, inspiratory muscle strength, and aerobic capacity in PDN with OSA on hypothesizing that IMT could be an effective modality for enhancing lung functions, inspiratory muscle strength, and aerobic capacity in PDN with OSA.

## 2. Methods

### 2.1. Subjects

A randomized controlled trial was conducted on 55 diabetic patients, their criteria according to the National Diabetes Data Group criteria [27], recruited from the Outpatient Clinic of Endocrinology department in the university hospital, Prince Sattam bin Abdulaziz University. Moderate DPN patients were selected in this study in keeping with the neuropathy symptom score (NSS) and neuropathy disability score (NDS) [28]. Also, patients were included in the study if their maximal inspiratory pressure (PImax) is less than or equal 70% predicted [29]. In addition, they were diagnosed with mild to moderate OSA (AHI 5 to 30/h with clinical symptoms). Polysomnogram (PSG) was used for OSA diagnosis; sleep was assessed by one-night polysomnogram (PSG) that was performed by a Somon Medics Gmbh (Am SonnenstuhL63, D-97236 Rander acker, Germany, Type: SOMNO Screen™ Plus, SN: 4259, kw45: 2014). Thermal airflow sensors (thermistor) were used for respiratory sensors, nasal, and oral signals. Apneas and hypopneas are scored and added to determine the AHI [30]. Regarding the American Academy of Sleep Medicine definitions, an apnea was defined as ≥90% drop in the thermistor excursion signal of ≥10 sec and hypopnea which was defined as a decrease in thermistor signal by >30% for >10 sec accompanied by a decrease of oxyhemoglobin saturation ≥ 4%. The patient was considered to have sleep apnea syndrome (SAS) if the apnea hypopnea index (AHI) is ≥15 or if AHI ≥ 5 is associated with insomnia, cardiovascular comorbidities or diurnal symptoms mainly EDS, impaired cognition, or mood changes [31].

Patients with respiratory or cardiac problems; end-stage renal disease (ESRD); severe chronic liver disease; chronic pain; and advanced metabolic, neuropsychiatric, or endocrinal disorders affecting sleep; and BMI > 34 kg/m^2^ were excluded from the study. Also previous patients with a diagnosis of type 1 diabetes or the presence of an acute illness at study enrollment, history of medication intake affecting sleep, and who are heavy smokers or drug abusers were excluded. In addition, patients underwent regular aerobic exercise were not included in this study as it improves inspiratory muscle strength so only sedentary patients were recruited [32, 33].

This study was ethically approved by the local research ethical committee of the physical therapy department at Prince Sattam bin Abdulaziz University in reference to the standards of the Helsinki Declaration and CONSORT guidelines (No: RHPT/019/032). All patients were informed that their participation is voluntary and instructed to sign the consent form before beginning the study procedure.

### 2.2. Assessments

Medical history, body weight, and height were recorded for each participant. Body mass index (BMI) was calculated by dividing weight in kilograms on height square in meters. Subjects were instructed to come fasting for taking of the samples, and assessment of blood glucose and HbA1c were done. Medical history, physical examination, and the determination of PImax were done. Included patients were randomly assigned into the IMT and placebo IMT (P-IMT) groups. Lung functions, inspiratory muscle strength, and aerobic capacity were assessed before and after 12 weeks of the study period.

### 2.3. Inspiratory Muscle Strength

The values of maximal inspiratory pressures have been obtained using the POWERbreathe KH2 equipment, and the maximal inspiratory pressure (PImax) has been recorded in cmH_2_O. For PImax measurement, subjects generated a maximal inspiration from a residual lung volume and against a constant resistance. The maneuvers were carried out and the participants were instructed to sit with knees flexed to 90° and close the nose using the thumb and index fingers. A warm-up exercise was in the form of a complete approximately five breaths against resistance. Three measurements were obtained making sure that a maximum value was reached [34, 35].

### 2.4. Lung Functions

The Minispir® Light spirometer with Winspiro® Light software was utilized to measure lung function. Patients were seated with flexed knees at 90° and held three deep breaths, inspired up to the total lung capacity (TLC), and then exhaled all the air to their residual volume (RV) to obtain the variables FEV1 (forced expiratory volume in 1 s), FVC (forced vital capacity), and maximal voluntary ventilation (MVV) and FEV1/FVC. The test was repeated at least three times until the system considered the best maneuver as more reproducible and acceptable, considering the reference values for the adult Brazilian population [36].

### 2.5. Aerobic Capacity

Cardiopulmonary exercise test (CPET) was conducted using a treadmill (Centurium 300, Micromed, Brazil). The software ErgoPCElite® was associated with 12-lead electrocardiogram (Micromed, Brazil). Temperature (18–22°C) and humidity (50–70%) were the optimal condition for gathering the parameters; a gas analyzer is attached with a facemask during exercise. There was no verbal communication from the patients during the test, telling the patients to use hand signals to express fatigue level. The termination of the test was done by the monitor, or by the patients reporting about exhaustion [37].

### 2.6. Intervention

Both groups were recommended to maintain their regular activity daily livings and adhere to pulmonologist instructions. The program was supervised by professional physiotherapists in the physical therapy outpatient clinic.

An electronic inspiratory muscle trainer (TRAINAIR®, Project Electronics Ltd., UK) was connected to the computer and used for inspiratory muscle training. It consists of a computer biofeedback system with software and electronic pressure manometer. The pressure produced by it is about 0−300 cmH_2_O. The training of the patients was referred to as their capacity with a fixed load of 75% of PImax. Each training was consisted of six cycles of thirty breaths. Each cycle consisted of around 4 min of resisted breathing. The resting period was 1 min after the first cycle, 45 sec after the second and third cycles, and 40 sec in fourth and fifth cycles up to the sixth cycle. The total duration of the session was approximately 30 min, three times a week for 12 consecutive weeks. Breaths through the device were applied by wearing a nose clip [38]. The P-IMT group was trained at a low training intensity (≤10% of PImax) with the same exercise protocol.

### 2.7. Statistical Analysis

Data were statistically analyzed utilizing SPSS version 20 (IBM Corp, Armonk, NY, USA). The Shapiro-Wilk test was used to assess the normality of data distribution. Characteristics of the sample and treatment effect between groups were explored using the unpaired *t*-test, while the paired *t*-test was used to compare post mean values within each group. Analyzed data are shown as mean ± standard deviation. Statistical significance was set at *p* < 0.05.

## 3. Results

**Figure 1:**
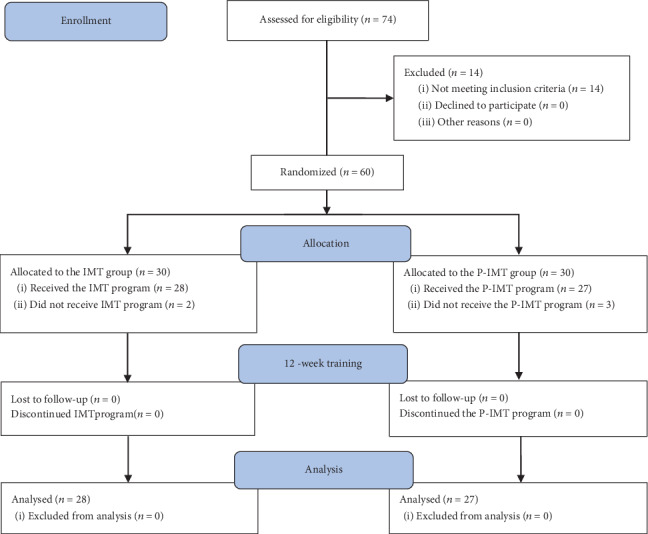
The flowchart of the study.

Figure 1 shows the flowchart of the current study. Of the 74 patients with PDN, 14 did not meet the inclusion criteria. 60 patients were randomized into the IMT (30) and P-IMT (30) groups; two patients from the IMT group and three patients from the P-IMT group later dropped out without defined reasons. Therefore, 28 patients from the IMT group and 27 patients from the P-IMT group completed the study and their results were analyzed postintervention.

As presented in Table 1, there were no significant differences between the groups regarding baseline demographic and clinical characteristics of the study (age, gender, BMI, HbA1c, AHI, lung functions, inspiratory muscle functions anthropometric, and cardiopulmonary exercise tests; *p* > 0.05) before training. Analyzing the after-training mean values, no significant changes were observed regarding lung functions in the IMT and P-IMT groups (*p* > 0.05), while it was detected that the IMT group shows greater improvement in FVC and FEV1 than the P-IMT group (*p* < 0.05). PImax improved in the IMT group (*p* < 0.05), while no significant changes were observed in the P-IMT group (*p* > 0.05). VO_2_max and VCO_2_max improved postintervention as compared with before training (*p* < 0.05) in the IMT group and did not change in the P-IMT group (*p* > 0.05). Other cardiopulmonary exercise testing variables showed no changes after training in the two groups (*p* > 0.05). Comparing between the IMT and P-IMT groups after training, there were significant differences between the two groups in FVC, FEV1, PImax, VO_2_max, SBP max, and DBP max (*p* < 0.05) in favor of the IMT group as detailed in Table 2.

## 4. Discussion

The current study was proposed to evaluate the effect of IMT on lung function and aerobic capacity in PDN with OSA. It is hypothesized that IMT is an effective method for improving lung function and aerobic capacity. The finding of this study showed improvements in inspiratory muscle strength and aerobic capacity without changes in the lung function in PDN with OSA. Also, we observed that PImax and PImax% were lower than the predicted values before intervention. This observation was supported by Kilicli et al. who approved that T2D is associated with lower values of PImax and PImax% [39]. Regarding lung functions, it has been reported that there were no changes in FEV1, FVC, and MVV because of normal lung functions at the beginning of the study.

Commonly, PDN and OSA lead to a deprived glycemic control and OSA is associated with a lack of aerobic capacity due to alterations of the sympathetic nervous system activity, hypothalamic-pituitary-adrenal axis, and formation of reactive oxygen species that lead to an increase of inflammatory cytokines, tumor necrosis factor, and adipocyte-derived factors [40, 41]. A prior study has approved that patients with T2D and OSA have impaired inspiratory muscle strength when compared with the matched reference value of non-OSA individuals [42]. In addition, it was demonstrated that the muscle strength of the upper airways is strongly correlated with inspiratory pumping muscle strength in OSA patients [43]. Also, hypoxemia and hypercarbia of the chemoreflex may increase the activity of the sympathetic nervous system that results in OSA-related comorbidities [44, 45].

In agreement with our findings, Correa et al. provided that IMT improves inspiratory muscle strength, aerobic capacity, and PImax values in T2D patients [26]. Also, it was reported that 4 weeks of IMT improves inspiratory muscle strength in OSA patients [46–48]. Furthermore, previous studies approved the positive effects of IMT on inspiratory muscle strength, functional status, diaphragmatic muscle performance, and circulation of the rest extremities [49, 50]. Moreover, similar to the current study results, it was documented that IMT improved PImax in healthy individuals through controlling the power breath during the endurance test of inspiratory muscles [51]. In addition, another study concluded that inspiratory and expiratory muscle training improves ventilator efficiency, inspiratory muscle strength, and aerobic capacity in normal individuals [52].

On the contrary, one study approved that a combination of aerobic exercise and IMT showed no efficient changes in aerobic capacity whether maximal oxygen uptake or other measures of the cardiopulmonary exercise test [53] that could result from the limited focus on respiratory muscles in comparison with aerobic exercise and/or the seminormal functional status of the included patients. Also, another study concluded that IMT improves inspiratory muscle strength but does not improve aerobic capacity in healthy older adults [54] which could be associated with the different baseline data.

It is believed that metaboreflex activation in inspiratory muscle leads to high blood flow to both the skeletal and inspiratory muscles in normal subjects [55] and chronic obstructive pulmonary disease patients [22]. On the other hand, diabetic patients had showed no improvement in aerobic capacity following IMT because of the muscle metaboreflex attenuation [56].

In spite of the strong relation between OSA and PDN, however, this relation is still not understood. Both of PDN and OSA lead to the inability to control the blood glucose level. Also, OSA is related to disturbed aerobic capacity while no studies confirmed these observations. Regarding the study results, inspiratory muscle strength and aerobic capacity had improved following IMT in patients suffering from PDN and OSA.

Some limitations were observed in the study. The main limitation is the short study period. Another limitation is that there were no intermediate and postintervention follow-up assessments. One more limitation is that the quality of life was not assessed postintervention. Future studies are needed to assess the effectiveness of IMT in diabetic patients with abnormal lung functions.

## 5. Conclusions

This randomized trial provided that IMT may improve inspiratory muscle strength and aerobic capacity in PDN with OSA. It also provides clinical implications of IMT in the treatment of PDN patients with OSA. IMT should be recommended in pulmonary rehabilitation programs, especially PDN patients with OSA.

## Figures and Tables

**Table 1:** Baseline demographic and clinical characteristics of the study groups.

Variables	IMT group (*n* = 28)	P-IMT group (*n* = 27)	*p* value
Age	55.5 ± 9.8	59.5 ± 4.8	0.061
Gender (M/F)	20/8	22/5	0.381
BMI (kg/m^2^)	29.2 ± 3.9	27.9 ± 4.8	0.274
HbA1c (%)	7.3 ± 1.6	7.2 ± 1.9	0.833
AHI (events)	32 ± 11.7	31 ± 10.8	0.743
Lung functions (% predicted)			
FEV1	105 ± 29	96 ± 13	0.146
FVC	103 ± 29	92 ± 10	0.067
MVV	80 ± 18	79 ± 17	0.833
Inspiratory muscle functions			
PImax (cm H_2_O)	56 ± 13	52 ± 10	0.207
PImax (% predicted)	58 ± 11	57 ± 10	0.726
Cardiopulmonary exercise test			
VO_2_max (ml/kg/min)	17.6 ± 3.4	19.4 ± 3.5	0.058
VCO_2_max (ml/kg/min)	29.6 ± 3.9	28.0 ± 7.1	0.303
HR rest (bpm)	76.9 ± 6.0	75.3 ± 6.7	0.354
HR max (bpm)	140.4 ± 11.0	141.5 ± 9.4	0.692
SBP rest (mmHg)	110.5 ± 10.4	111.3 ± 6.9	0.739
SBP max (mmHg)	171.3 ± 15.6	178.8 ± 17.3	0.097
DBP rest (mmHg)	69.7 ± 6.5	68.1 ± 4.2	0.285
DBP max (mmHg)	87.5 ± 7.1	90.0 ± 9.3	0.266

IMT: inspiratory muscle training; P-IMT: placebo-inspiratory muscle training; BMI: body mass index; HbA1c: glycosylated hemoglobin; AHI: apnoea-hypopnoea index.

**Table 2:** The differences in the mean values of all outcome measures before and after training in the two groups.

Variables	IMT (*n* = 28)			P-IMT (*n* = 27)		
	Pre	Post	*p* value	Pre	Post	*p* value
Lung functions (% predicted)						
FEV1	105 ± 29	107 ± 18	0.757	96 ± 13	91 ± 17^∗^	0.231
FVC	103 ± 29	106 ± 18	0.643	92 ± 10	88 ± 13^∗^	0.211
MVV	80 ± 18	82 ± 13	0.635	79 ± 17	76 ± 14	0.482
Inspiratory muscle functions						
PImax (cmH_2_O)	56 ± 13	121 ± 22	<0.001	52 ± 10	57 ± 11^∗^	0.086
PImax (% predicted)	58 ± 11	125 ± 17	<0.001	57 ± 10	61 ± 9^∗^	0.128
Cardiopulmonary exercise test						
VO_2_max (ml/kg/min)	17.6 ± 3.4	24.7 ± 6.4	<0.001	19.4 ± 3.5	20.1 ± 2.4^∗^	0.395
VCO_2_max (ml/kg/min)	29.6 ± 3.9	29.1 ± 3.3	0.607	28.0 ± 7.1	27.0 ± 6.1	0.581
HR rest (bpm)	76.9 ± 6.0	75.3 ± 5.5	0.303	75.3 ± 6.7	74.3 ± 7.2	0.599
HR max (bpm)	140.4 ± 11.0	143.6 ± 10.6	0.273	141.5 ± 9.4	143.8 ± 9.9	0.385
SBP rest (mmHg)	110.5 ± 10.4	108.3 ± 12.9	0.485	111.3 ± 6.9	110.5 ± 7.6	0.687
SBP max (mmHg)	171.3 ± 15.6	167.0 ± 12.7	0.263	178.8 ± 17.3	182.5 ± 15.8^∗^	0.416
DBP rest (mmHg)	69.7 ± 6.5	67.8 ± 5.8	0.253	68.1 ± 4.2	66.4 ± 6.9	0.279
DBP max (mmHg)	87.5 ± 7.1	85.0 ± 5.3	0.141	90.0 ± 9.3	92.5 ± 8.9^∗^	0.317

^∗^Significant difference between groups postintervention (*p* < 0.05). IMT: inspiratory muscle training; P-IMT: placebo-inspiratory muscle training; FEV1: forced expiratory volume in 1 sec; FVC: forced vital capacity; MVV: maximal voluntary ventilation; PImax: maximum inspiratory pressure; VO_2_max: maximal oxygen consumption; VCO_2_max: maximal carbon dioxide exhaled; HRmax: maximal heart rate; SBP: systolic blood pressure; DBP: diastolic blood pressure.

## Data Availability

Regarding manuscript 5036585, this study is a randomized control study and the data involved is available from the corresponding author upon request and privacy-related parts of the patient will not be provided.
